# Salivary Methotrexate as a Predictor of Oral Mucositis in Hematological Cancer Patients: A Phase II Randomized, Double‐Blind Clinical Trial

**DOI:** 10.1111/odi.70381

**Published:** 2026-07-12

**Authors:** Letícia Rodrigues‐Oliveira, Carolina Guimarães Bonfim, Ana Leticia Mores, Natalia Rangel Palmier, Henrique Ballassini Abdalla, Juliana Trindade Clemente‐Napimoga, Iara Gonçalves Aquino, Maria Cecilia Querido de Oliveira, Maria Cláudia Cuzzullin, Aljomar Jose Vechiato Filho, Cesar Rivera, Tatiana Natasha Toporcov, Juliana Pereira, Luiz Alcino Gueiros, Rossana Veronica Mendoza López, Manoela Domingues Martins, Marcelo Lazzaron Lamers, Karina Morais‐Faria, Pablo Agustin Vargas, Alan Roger Santos‐Silva, Thais Bianca Brandão, Ana Carolina Prado‐Ribeiro

**Affiliations:** ^1^ Oral Diagnosis Department, Faculdade de Odontologia de Piracicaba Universidade de Campinas (UNICAMP) Piracicaba São Paulo Brazil; ^2^ Dental Oncology Service, Instituto Do Câncer Do Estado de São Paulo Faculdade de Medicina da Universidade de São Paulo (ICESP‐FMUSP) São Paulo Brazil; ^3^ Faculdade São Leopoldo Mandic Campinas São Paulo Brazil; ^4^ Laboratorio de Histopatología Oral y Maxilofacial, Unidad de Medicina Oral y Patología Oral, Departamento de Estomatología, Facultad de Odontología Universidad de Talca Talca Región del Maule Chile; ^5^ Departamento de Epidemiologia, Faculdade de Saúde Pública Universidade de São Paulo São Paulo São Paulo Brazil; ^6^ Discipline of Hematology University of São Paulo São Paulo Brazil; ^7^ Department of Clinic and Preventive Dentistry Federal University of Pernambuco Recife Pernambuco Brazil; ^8^ National Institute of Science and Technology in Translational Biophotonics in Dentistry (INCT‐BIOFOTO BUCAL) Porto Alegre Brazil; ^9^ Comprehensive Center for Precision Oncology C2PO, Center for Translational Research in Oncology, Instituto do Câncer do Estado de São Paulo Faculdade de Medicina da Universidade de São Paulo (ICESP‐FMUSP) São Paulo Brazil; ^10^ Department of Oral Pathology, School of Dentistry Federal University of Rio Grande do Sul Porto Alegre Rio Grande do Sul Brazil; ^11^ Basic Research Center in Dentistry, Dentistry School Federal University of Rio Grande do Sul Porto Alegre Brazil; ^12^ Department of Morphological Sciences, Institute of Basic Health Sciences Federal University of Rio Grande do Sul Porto Alegre Brazil

**Keywords:** cancer, low‐level laser therapy, methotrexate, oral mucositis

## Abstract

**Objective:**

To evaluate the efficacy of prophylactic photobiomodulation therapy (PBMT) in preventing oral mucositis (OM) and to assess serum and salivary methotrexate (MTX) levels as predictors of OM in adults receiving high‐dose methotrexate (HD‐MTX).

**Methods:**

This phase II randomized, double‐blind, placebo‐controlled trial enrolled adults with hematological malignancies undergoing a first HD‐MTX cycle at a tertiary cancer center in Brazil. Participants were randomized to PBMT or sham laser therapy, administered daily until MTX clearance or for up to five sessions. OM was assessed for 9 days using WHO criteria. Serum and salivary MTX concentrations were measured at 24, 48, and 72 h post‐infusion, along with urinary pH and serum creatinine.

**Results:**

Fifty‐three patients were randomized (PBMT *n* = 26; placebo *n* = 27). PBMT significantly reduced ulcerative OM (grade ≥ 2) at 96 h (11.5% vs. 51.9%), yielding a 77.7% relative risk reduction and a number needed to treat of 2 (*p* < 0.01). Placebo recipients had an 8.97‐fold higher odds of severe OM. Serum MTX was not associated with OM, whereas salivary MTX increased disproportionately over time and independently correlated with OM severity.

**Conclusions:**

PBMT significantly reduced OM incidence and severity in adults receiving HD‐MTX. Salivary MTX is a promising non‐invasive biomarker for OM risk.

**Trial Registration:**

Trial was prospectively registered in the Brazilian Registry of Clinical Trials (ReBec, No. 12218)

## 1 Introduction

High‐dose methotrexate (HD‐MTX, ≥ 1 g/m^2^) is widely utilized in adults and children as a chemotherapeutic agent for the treatment of solid tumors and hematological malignancies with suspected or proven central nervous system (CNS) involvement due to its ability to penetrate the blood–brain barrier (Calimeri and Ferreri 2020; Ferrari et al. 1993; Rask et al. 1998; Thomas et al. 1999; Wight et al. 2022; Wilson et al. 2020). It remains a cornerstone in oncology, particularly for lymphomas and osteosarcomas (Calimeri and Ferreri 2020; Ferrari et al. 1993; Wight et al. 2022; Wilson et al. 2020).

Methotrexate (MTX), a folate antagonist, inhibits dihydrofolate reductase and thymidylate synthase, disrupting DNA and RNA synthesis and impairing cellular proliferation. HD‐MTX affects not only neoplastic cells, but also normal tissues with high proliferative rates (Wight et al. 2022; Howard et al. 2016; Hu et al. 2024; Valer et al. 2021; Van der Beek et al. 2019). Toxicity correlates with dose, exposure time, and elevated plasma MTX levels and includes myelosuppression, oral mucositis (OM), acute kidney injury, and hepatotoxicity (Rask et al. 1998; Wight et al. 2022; Howard et al. 2016; Oliff et al. 1979; Den Hoed et al. 2015; Liu et al. 2011).

OM is a particularly burdensome complication of HD‐MTX, affecting the oral and gastrointestinal mucosa with erythema, erosions, and ulcerations. It causes pain, reduced intake, weight loss, and impaired quality of life, often leading to hospitalizations, increased costs, and treatment delays that compromise prognosis and survival (Van der Beek et al. 2019; Rodrigues‐Oliveira et al. 2021).

Reported OM incidence in adults receiving HD‐MTX ranges from 6% to 15%, underscoring the need for effective preventive strategies (Wight et al. 2022; Wilson et al. 2020; Shenoy et al. 2022). Photobiomodulation therapy (PBMT) enhances tissue repair and reduces pain and has gained wide acceptance for the prevention and management of OM (Heimlich et al. 2023). Clinical guidelines and systematic reviews highlight the fundamental role of prophylactic PBMT in improving OM‐related outcomes; however, to date, no randomized trial has specifically evaluated preventive PBMT in adults with hematological malignancies undergoing HD‐MTX (Elad et al. 2020; Zadik et al. 2019).

This study therefore primarily evaluated whether prophylactic PBMT reduces the incidence and severity of OM in adults treated with HD‐MTX and secondarily assessed whether salivary MTX levels are associated with OM severity.

## 2 Methods

### 2.1 Study Design and Setting

This prospective, randomized, double‐blind, controlled clinical trial conducted at the Instituto do Câncer do Estado de São Paulo (ICESP), São Paulo, Brazil, between 2022 and 2024. Ethical approval was obtained (CAAE: 38194420.4.0000.5418), and the trial was prospectively registered in the Brazilian Registry of Clinical Trials (ReBec, No. 12218). The protocol adhered to the Declaration of Helsinki (World Medical Association 2013) and followed the CONSORT 2010 (Moher et al. 2010) recommendations for randomized controlled trials. All participants provided written informed consent prior to enrollment.

### 2.2 Eligibility Criteria

Inclusion criteria: patients aged ≥ 18 years, diagnosed with hematological malignancies, and eligible to receive intravenous HD‐MTX for the first time.

Exclusion criteria: refusal or lack of consent to participate; HD‐MTX administration prior to dental assessment; cognitive impairment interfering with study procedures; admission to the intensive care unit (ICU); participation in other clinical trials; or death before initiation of the intervention.

### 2.3 Randomization, Allocation Concealment, and Blinding

Patients were randomized in a 1:1 ratio using permuted blocks of four participants (block size = 4; Microsoft Excel (Microsoft Corporation 2023)) to prophylactic PBMT (Group A) or active placebo/sham laser (Group B). No stratification or balancing factors were used in the allocation process. The allocation sequence was not accessible to patients or clinical evaluators, who remained blinded to group assignment throughout the trial; only the PBMT operator had access to the randomization list. Both groups underwent identical procedures, with Group B receiving sham irradiation using the same device with the laser switched off.

#### 2.3.1 Sample Size Calculation

The sample size was calculated a priori based on the primary endpoint, incidence of OM. Baseline incidence of 0.71 and the expected reduction to 0.29 were informed by data from De Castro et al. (2013). Using these estimates, 52 participants (26 per group) were required to achieve 80% power with a two‐sided *α* = 0.05.

#### 2.3.2 Data Collection and Management

Baseline demographic and clinical data were obtained through structured interviews or extracted from electronic medical records (Philips Healthcare 2023). All data were entered into REDCap (Harris et al. 2009; Vanderbilt University 2023) (v14.0.16) in accordance with the Brazilian General Personal Data Protection Law (LGPD; Law No. 13,709/2018).

### 2.4 Interventions

Standard oral care consisted of daily 0.12% chlorhexidine mouthwash, oral hygiene and nutritional guidance, and counseling regarding HD‐MTX‐related toxicities. All treatment procedures are summarized in Figure 1, and PBMT parameters are detailed in Table S1.

**FIGURE 1:**
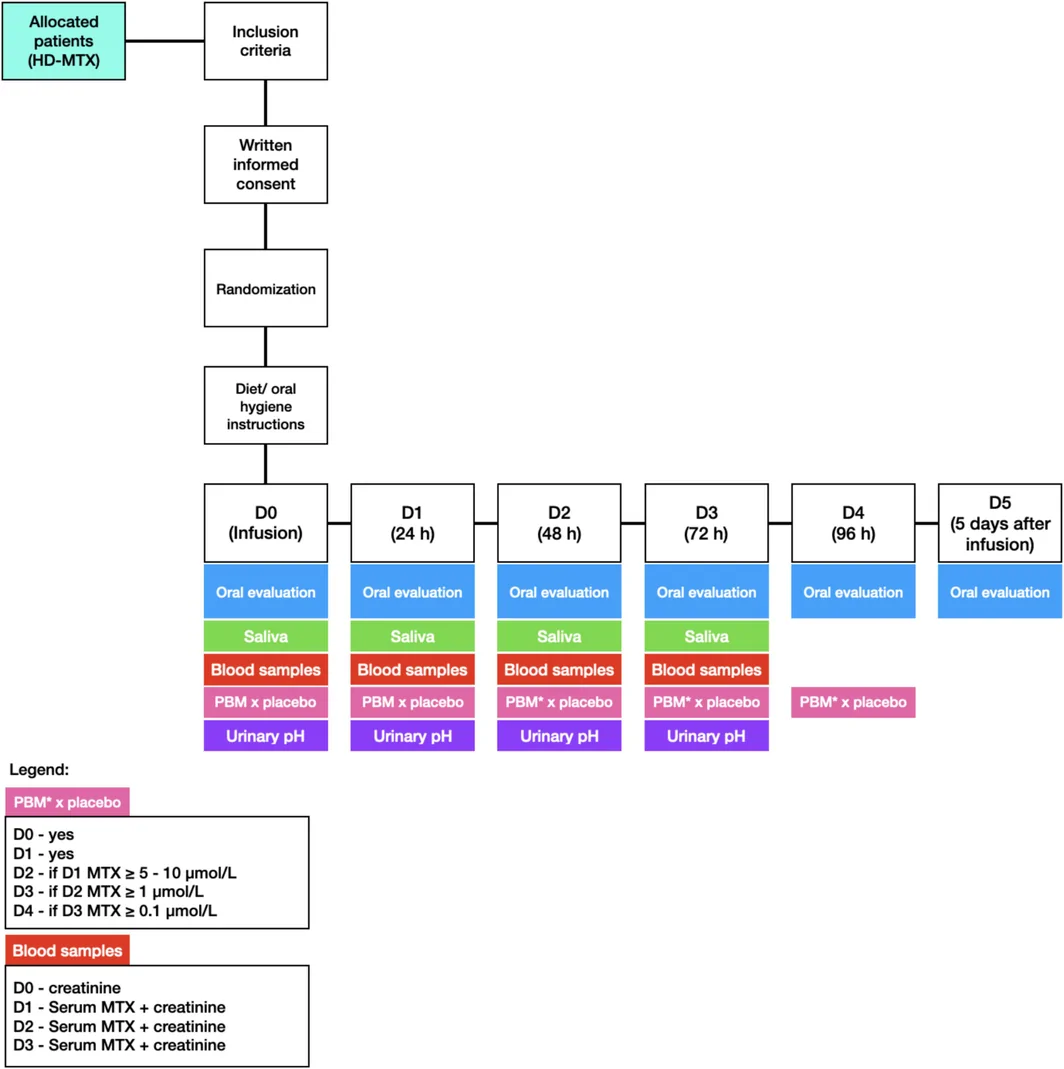
Flow chart of patient's follow‐up and procedures during the study. PBM* photobiomodulation.

#### 2.4.1 PBMT Protocols

Group A (PBMT): irradiation of the oral commissures (1 point), labial mucosa (3), buccal mucosa (3), lateral tongue borders (3), ventral tongue (2), anterior floor of mouth (2), and soft palate (2), following Brandão et al. (2018).

Group B (Sham): identical procedures with the laser switched off.

All procedures were performed by the same trained dentist.

### 2.5 Clinical and Laboratory Monitoring

#### 2.5.1 Hematological Parameters

All patients underwent a complete blood count prior to initiating HD‐MTX. Leukocyte counts were recorded, and abnormal values were defined as < 4000/mm^3^ or > 11,000/mm^3^ based on standard laboratory reference ranges.

#### 2.5.2 Serum MTX Level

Plasma MTX concentrations were measured on days 1–3 (D1–D3) post‐HD‐MTX. Delayed excretion was defined as ≥ 5 μmol/L at 24 h, ≥ 0.5 μmol/L at 48 h, and ≥ 0.05 μmol/L at 72 h (Joannon et al. 2004). Serum MTX was quantified using a chemiluminescent microparticle immunoassay (2P49 ARCHITECT Methotrexate assay, Abbott GmbH, Germany).

#### 2.5.3 Renal Function and Urinary pH

Creatinine was assessed pre‐infusion and on D1–D3. Reference ranges were 0.70–1.20 mg/dL (men) and 0.50–0.90 mg/dL (women) (Jameson et al. 2018).

Urinary pH was measured at baseline and D1–D3 using dipsticks. Normal values are 4.5–8.0; ≥ 7.0 was recommended prior to HD‐MTX to minimize nephrotoxicity (Widemann and Adamson 2006).

#### 2.5.4 Saliva Collection and Analysis

Unstimulated saliva was collected on D1–D3 after ≥ 1 h fasting, over 5 min, and stored at −80°C. MTX was quantified by enzyme‐linked immunosorbent assay—ELISA (Human Methotrexate ELISA Kit, Bioassay Technology Laboratory, China) (Jaedicke et al. 2012).

### 2.6 Outcomes

The primary outcome was the incidence and severity of OM, assessed using the WHO Oral Mucositis Scale (grade 0 = no OM; grade 1 = erythema and soreness; grade 2 = erythema, ulcers, able to eat solids; grade 3 = ulcers, requires a liquid diet due to OM; grade 4 = ulcers, alimentation not possible due to OM) (Parulekar et al. 1998). Evaluators were appropriately calibrated, and clinical assessments were performed at baseline, 0, 24, 48, 72, and 96 h, and on day 9 following MTX infusion.

The primary endpoint was defined as the proportion of participants developing ulcerative OM (WHO grade ≥ 2) within 9 days after HD‐MTX infusion. Secondary clinical outcomes included the maximum OM grade, the trajectory of OM severity from 24 h to day 9 post‐infusion, the time‐to‐onset of OM, and patient‐reported symptoms such as dysgeusia, dysphagia, and xerostomia, assessed according to CTCAE v5.0 (U.S. Department of Health and Human Services 2017).

In addition to these clinical secondary outcomes, serum and salivary MTX concentrations, serum creatinine levels, and urinary pH were prespecified as exploratory laboratory endpoints, intended to (a) characterize MTX pharmacokinetics and elimination patterns and (b) explore potential associations with the development, severity, and time‐to‐onset of OM.

### 2.7 Statistical Analysis

Analyses followed a per‐protocol approach. Categorical variables were summarized as frequencies, and quantitative variables as means, standard deviations, medians, and ranges. Associations between categorical variables were evaluated using Fisher's exact test. Repeated measures were analyzed using linear mixed‐effects models, with model assumptions assessed through residual diagnostics, as described previously (Schall 1991). Post‐hoc analyses were performed using orthogonal contrasts. Associations between quantitative variables were assessed using linear or mixed‐effects regression models. OM severity was analyzed using multinomial logistic regression, including repeated‐measures extensions when appropriate (Agresti 1996; Hedeker and Gibbons 1994). Relative risks were estimated using Poisson regression with robust variance (Zou 2004). All analyses were performed in R (v4.1.3) and SAS (v9.4) (R Core Team 2023; SAS Institute 2013; Hosmer and Lemeshow 2000; Mehta and Patel 1983). Statistical significance was set at *α* = 0.05.

## 3 Results

### 3.1 Patient Characteristics and Treatment Data

#### 3.1.1 Eligibility and Study Population

Of 100 patients referred for HD‐MTX‐containing chemotherapy, 61 met eligibility criteria; 39 were excluded due to non‐compliance (*n* = 13; 33.33%), cognitive impairment/ICU admission (*n* = 10; 25.64%), non‐hematologic diagnosis (*n* = 6; 15.38%), delayed referral (*n* = 4; 10.26%), death before treatment (*n* = 4; 10.26%), or protocol change/other trial participation (*n* = 2; 5.13%). Among the 61 eligible, 7 (11.5%) missed appointments, and 1 (1%) was transferred to the ICU, leaving 53 patients randomized to Group A (PBMT, *n* = 26; 49.06%) or Group B (placebo/sham laser, *n* = 27; 50.94%) (Figure S1).

#### 3.1.2 Demographic Characteristics

Most patients were male (*n* = 34; 64.1%), with a mean age of 40.8 years (range 18–73), comparable between Group A (39.73) and Group B (41.78).

Lymphoma was the most frequent diagnosis (*n* = 36; 67.92%; Group A: *n* = 17; 65.38%; Group B: *n* = 19; 70.37%), followed by leukemia (*n* = 16; 30.19%; Group A: *n* = 8; 30.77%; Group B: *n* = 8; 29.63%) and blastic plasmacytoid dendritic cell neoplasm (*n* = 1; 1.89%; Group A: *n* = 1; 3.85%). Nine patients (16.98%) were HIV‐positive, evenly distributed.

Abnormal leukocyte counts were similarly distributed between groups (Group A: *n* = 15, 57.7%; Group B: *n* = 15, 55.6%). Although patients with abnormal leukocyte counts had a 2.31‐fold higher likelihood of developing more severe mucositis, this association was not statistically significant (*p* > 0.05).

#### 3.1.3 Treatment Protocols and HD‐MTX Administration

All patients received the first HD‐MTX cycle, as monotherapy or combination therapy. The most commonly used protocols were: prophylactic HD‐MTX for patients at high risk of central nervous system relapse (*n* = 15; 28.30%; Group A: *n* = 9; 34.62%; Group B: *n* = 6; 22.22%), CODOX‐M (*n* = 8; 15.09%; Group A: *n* = 4; 15.38%; Group B: *n* = 4; 14.81%) and GRAAPH 2005 (*n* = 7; 13.21%; Group A: *n* = 2; 7.69%; Group B: *n* = 5; 18.52%). Most infusions were outpatient (Group A: *n* = 19; 73.08%; Group B: *n* = 15; 55.56%). The overall mean infusion dose was 4455 mg/m^2^ (SD 2569.03, range 1560–14,880 mg/m^2^). Mean infusion time was 6.3 h (SD 5.9, range 2–24), similar across groups (Table 1).

**TABLE 1:** Summaries of patient characteristics, treatment information and HD‐MTX data.

Characteristics	Group A *n = 26* (49.06%)	Group B *n* = 27 (50.94%)	Total *n* = 53 (100%)
Gender			
Female	10 (38.46%)	9 (33.33%)	19 (35.85%)
Male	16 (61.54%)	18 (66.67%)	34 (64.15%)
Age (years)			
Mean (Standard Deviation)	39.73 (15.59)	41.78 (15.99)	40.77 (15.68)
Median (Minimum; Maximum)	39.00 (18.00; 73.00)	44.00 (20.00; 68.00)	41.00 (18.00; 73.00)
Hematological diseases			
Lymphoma	17 (65.38%)	19 (70.37%)	36 (67.92%)
Leukemia	8 (30.77%)	8 (29.63%)	16 (30.19%)
Blastic plasmacytoid dendritic cell Neoplasm	1 (3.85%)	0 (0.00%)	1 (1.89%)
Diagnoses			
HIV	4 (15.38%)	5 (18.52%)	9 (16.98%)
Syphilis	2 (7.69%)	4 (14.81%)	6 (11.32%)
Tuberculosis	1 (3.85%)	1 (3.70%)	2 (3.77%)
Diabetes	1 (3.85%)	2 (7.41%)	3 (5.66%)
Smoking habits			
No smoker	21 (80.77%)	18 (66.67%)	39 (73.58%)
Current smoker	1 (3.85%)	3 (11.11%)	4 (7.55%)
Former smoker	4 (15.38%)	6 (22.22%)	10 (18.87%)
Drinking habits			
No drinker	19 (73.08%)	20 (74.07%)	39 (73.58%)
Former drinker	1 (3.85%)	4 (14.81%)	5 (9.43%)
Social drinker	1 (3.85%)	0 (0.00%)	1 (1.89%)
Former social drinker	5 (19.23%)	3 (11.11%)	8 (15.09%)
Use of illicit drugs			
No	25 (96.15%)	25 (92.59%)	50 (94.34%)
Ex	1 (3.85%)	2 (7.41%)	3 (5.66%)
HD‐MTX setting			
Outpatient	*19 (73.08%)*	15 (55.56%)	34 (64.15%)
Inpatient	7 (26.92%)	12 (44.44%)	19 (35.85%)
Chemotherapy protocol			
Modified BFM 86	4 (15.38%)	2 (7.41%)	6 (11.32%)
BRALLA	1 (3.85%)	1 (3.70%)	2 (3.77%)
CODOX‐M	4 (15.38%)	4 (14.81%)	8 (15.09%)
COPADM1	1 (3.85%)	2 (7.41%)	3 (5.66%)
CYM‐1	0 (0.00%)	1 (3.70%)	1 (1.89%)
GRAAPH 2005	2 (7.69%)	5 (18.52%)	7 (13.21%)
Hyper CVAD	3 (11.54%)	0 (0.00%)	3 (5.66%)
CNS prophylaxis (MTX 3 g/m^2^)	9 (34.62%)	6 (22.22%)	15 (28.30%)
R‐CHOMP	2 (7.69%)	2 (7.41%)	4 (7.55%)
R‐MVP	0 (0.00%)	4 (14.81%)	4 (7.55%)
MTX dose (mg)			
Mean (Standard Deviation)	4822.88 (2394.41)	4102.04 (2724.42)	4455.66 (2569.03)
Median (Minimum; Maximum)	5535.00 (1570.00; 12720.00)	3000.00 (1560.00; 14880.00)	4650.00 (1560.00; 14880.00)
Duration of MTX infusion (hour)			
Mean (Standard Deviation)	6.68 (6.47)	5.86 (5.44)	6.27 (5.92)
Median (Minimum; Maximum)	4.38 (2.00; 24.00)	4.33 (2.00; 24.00)	4.33 (2.00; 24.00)

*Note:* BRALLA, doxorubicin, vincristine, daunorubicin, pegaspargase, cyclophosphamide, cytarabine, mercaptopurine, prednisone, and HD‐MTX; CNS prophylaxis, HD‐MTX; CNS, central nervous system; CODOX‐M, doxorubicin, vincristine, cyclophosphamide, cytarabine, and HD‐MTX; COPADM1, dexamethasone, vincristine, doxorubicin, cyclophosphamide, and HD‐MTX; CYM‐1, dexamethasone, cytarabine, HD‐MTX; GRAAPH 2005, dexamethasone, imatinib, vincristine, *HD‐MTX*, *cytarabine*; HD‐MTX, high‐dose methotrexate; Hyper CVAD, cyclophosfamide, vincristine, doxorubicin, dexamethasone, cytarabine, HD‐MTX; Modified BFM 86, dexamethasone, mercaptopurine, HD‐MTX; R‐CHOMP, rituximab, cyclophosphamide, doxorubicin, vincristine, prednisone, and HD‐MTX; R‐MVP, rituximab, vincristine, HD‐MTX.

Supportive care (hydration, urinary alkalinization, folinic acid rescue) followed institutional protocols.

### 3.2 MTX Concentration Monitoring (Serum and Saliva), Creatinine, and Urinary pH

Serum and salivary MTX levels, delayed elimination rates, creatinine, and urinary pH are summarized in Table 2, and Figures 2 and 3.

**TABLE 2:** Methotrexate excretion in blood and saliva at 24 h, 48 h, and 72 h.

	Group A *n* = 26 (49.06%)	Group B *n* = 27 (50.94%)	Total *n* = 53 (100%)
Blood (μmol/L)			
24 h			
Mean (Standard Deviation)	1.19 (1.01)	2.61 (4.33)	1.91 (3.22)
Median (Minimum; Maximum)	0.83 (0.16; 4.75)	1.07 (0.06; 17.71)	0.83 (0.06; 17.71)
% of delayed excretion	0 (0.00%)	5 (18.52%)	5 (9.43%)
48 h			
Mean (Standard Deviation)	0.09 (0.06)	0.23 (0.32)	0.16 (0.24)
Median (Minimum; Maximum)	0.07 (0.00; 0.26)	0.08 (0.00; 1.44)	0.08 (0.00; 1.44)
% of delayed excretion	0 (0.00%)	4 (14.81%)	4 (7.55%)
72 h			
Mean (Standard Deviation)	0.02 (0.03)	0.08 (0.14)	0.05 (0.10)
Median (Minimum; Maximum)	0.03 (0.00; 0.10)	0.02 (0.00; 0.62)	0.02 (0.00; 0.62)
% of delayed excretion	5 (19.23%)	10 (37.04%)	15 (28.30%)
Saliva (μmol/L)			
24 h			
Mean (Standard Deviation)	0.16 (0.13)	0.11 (0.09)	0.14 (0.11)
Median (Minimum; Maximum)	0.12 (0.00; 0.53)	0.12 (0.00; 0.42)	0.12 (0.00; 0.53)
48 h			
Mean (Standard Deviation)	0.12 (0.10)	0.26 (0.42)	0.19 (0.31)
Median (Minimum; Maximum)	0.10 (0.00; 0.37)	0.16 (0.00; 2.01)	0.13 (0.00; 2.01)
72 h			
Mean (Standard Deviation)	0.17 (0.37)	0.12 (0.09)	0.14 (0.26)
Median (Minimum; Maximum)	0.09 (0.00; 1.95)	0.12 (0.00; 0.31)	0.11 (0.00; 1.95)

**FIGURE 2:**
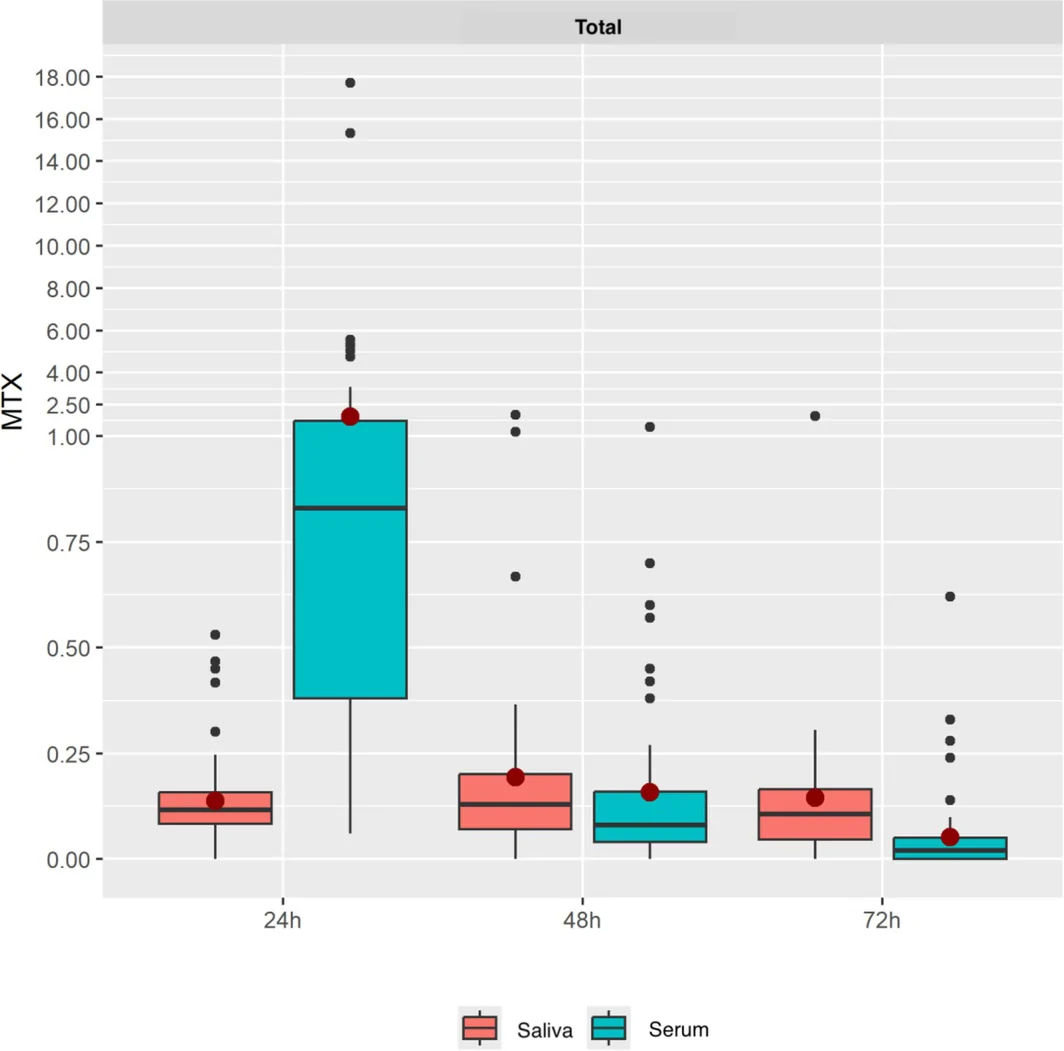
Serum and salivary MTX concentrations at 24 h, 48 h, and 72 h after HD‐MTX infusions. MTX* μmol/L.

**FIGURE 3:**
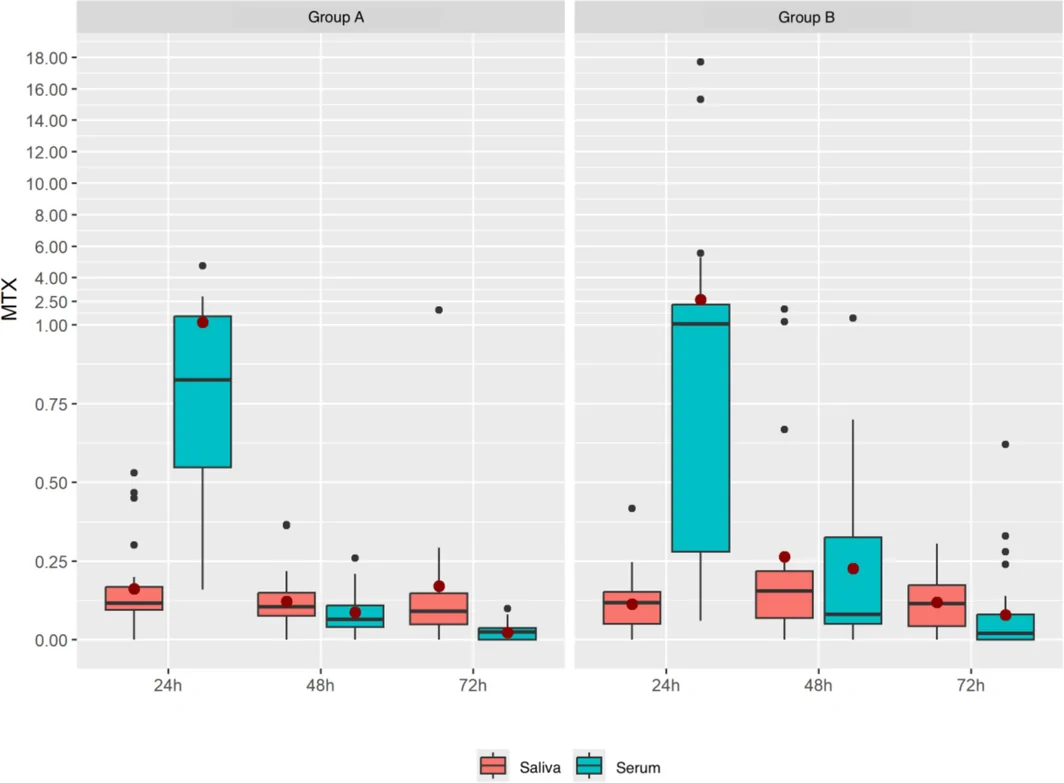
Serum and salivary MTX concentrations at 24 h, 48 h, and 72 h after HD‐MTX infusion in Group A and Group B. MTX* μmol/L.

#### 3.2.1 Serum‐MTX Excretion

Serum MTX declined biphasically. Delayed elimination occurred only at 72 h in Group A (*n* = 5; 19.23%), whereas in Group B, delayed elimination was detected at 24 h (*n* = 5; 18.52%), 48 h (*n* = 4; 14.81%), and 72 h (*n* = 10; 37.04%).

#### 3.2.2 Salivary‐MTX Excretion

Salivary MTX showed variable patterns. In Group A, concentrations were lowest at 48 h (0.12 ± 0.10 μmol/L) compared to 24 h (0.16 ± 0.13 μmol/L) and 72 h (0.17 ± 0.37 μmol/L). In contrast, Group B demonstrated higher excretion at 48 h (0.26 ± 0.42 μmol/L), with lower values at 24 h (0.11 ± 0.09 μmol/L) and 72 h (0.12 ± 0.09 μmol/L). When both groups were analyzed, salivary MTX peaked at 48 h (0.19 ± 0.31 μmol/L), while values at 24 and 72 h were similar (0.14 μmol/L, respectively). Across all time points, mean salivary MTX concentrations remained similar at 24 and 72 h, respectively.

Salivary MTX corresponded to 29%, 323%, and 589% of serum levels at 24 h, 48 h, and 72 h, respectively, with no significant serum–saliva correlation. Excretion rates by category are presented in Table S2.

### 3.3 Creatinine and Urinary pH

Abnormal serum creatinine occurred at 24 h–72 h in both groups (Table S3) with no correlation to serum MTX levels.

Most patients started HD‐MTX with urinary pH ≥ 7, except one (1.89%, Group A). Mean and standard deviation pH for each group at all evaluation times are shown in Table S3.

Mean urinary pH was lower in Group A. In Group A, a 1‐unit pH increase at 24 h was associated with higher serum MTX at 48 h (Δ = 0.0363, *p* = 0.04), whereas in Group B, a 1‐unit increase at 48 h corresponded to lower salivary MTX at 72 h (Δ = –0.0517, *p* = 0.047).

### 3.4 Oral Mucositis (OM)

OM occurred in 83.02% (*n* = 44) of patients—69.23% (*n* = 17) in Group A and 96.3% (*n* = 26) in Group B (Table 3). Time to onset was similar; grade 1 erythema appeared at 24 h and grade 2 ulcers at 48 h, with no association between OM ≥ 2 and group (*p* = 0.76).

**TABLE 3:** Oral mucositis in group A, group B, both groups at 24 h, 48 h, 72 h, 96 h, and 9 days post infusion.

	Group A *n* = 26 (49.06%)	Group B *n* = 27 (50.94%)	Total *n* = 53 (100%)
Oral mucositis*			
D1 (24 h post MTX infusion)			
Grade 0	20 (76.92%)	20 (74.07%)	40 (75.47%)
Grade 1	6 (23.08%)	7 (25.93%)	13 (24.53%)
D2 (48 h post MTX infusion)			
Grade 0	11 (42.31%)	10 (37.04)	21 (39.62%)
Grade 1	14 (53.85%)	14 (51.85%)	28 (52.83%)
Grade 2	1 (3.85%)	3 (11.11%)	4 (7.55%)
D3 (72 h post MTX infusion)			
Grade 0	12 (46.15%)	7 (25.93%)	19 (35.85%)
Grade 1	12 (46.15%)	14 (51.85%)	26 (49.06%)
Grade 2	2 (7.69%)	5 (18.52%)	7 (13.21%)
Grade 3	0 (0.00%)	1 (3.70%)	1 (1.89%)
D4 (96 h post MTX infusion)			
Grade 0	15 (57.69%)	4 (14.81%)	19 (35.85%)
Grade 1	8 (30.77%)	9 (33.33%)	17 (32.08%)
Grade 2	3 (11.54%)	12 (44.44%)	15 (28.30%)
Grade 3	0 (0.00%)	2 (7.41%)	2 (3.77%)
D5 (09 days post MTX infusion)			
Grade 0	24 (92.31%)	21 (77.78%)	45 (84.91%)
Grade 1	1 (3.85%)	1 (3.70%)	2 (3.77%)
Grade 2	1 (3.85%)	3 (11.11%)	4 (7.55%)
Grade 3	0 (0.00%)	2 (7.41%)	2 (3.77%)

* WHO classification.

The distribution of OM grades was as follows: grade 1 occurred in 14 patients in Group A (53.85%) and 10 in Group B (37.04%); grade 2 occurred in 4 patients in Group A (15.38%) and 14 in Group B (51.85%); and grade 3 occurred only in Group B (*n* = 2; 7.41%). No grade 4 OM was observed. Eight patients in Group A (30.77%) and one in Group B (3.70%) did not develop OM (grade 0).

At 48 h, ulcerated OM affected 7.55% overall (Group A 3.85%, Group B 11.11%). At 96 h more than half of patients in Group B (*n* = 14; 51.85%) had ulcerated mucositis, compared to 11.54% (*n* = 3) in Group A. By day 9, 84.91% (*n* = 45) of patients had recovered to grade 0 (Group A *n* = 24; 92.31%; Group B *n* = 21; 77.78%), while grade 2–3 persisted in 17 patients (32.1% overall, 11.5% in Group A and 51.8% in group B).

The effect size of PBMT on ulcerated OM (grade ≥ 2) was clinically relevant. At 96 h, the relative risk reduction was 77.7% (95% CI 31.5%–92.8%), absolute risk reduction was 40.3% (95% CI 15.3%–59.3%). This corresponds to leading to a number need to treat equal to 2 (IC 95% 7–2), indicating that treating just two patients with PBMT would prevent one case of ulcerated OM.

### 3.5 Regression Analysis

Multinomial logistic regression confirmed that Group B patients had 8.97‐fold higher odds of higher OM grades than Group A (95% CI: 2.66–30.22; *p* < 0.01). Poisson regression indicated a nearly four‐fold increased risk of ulcerated OM (grade 2–3) in Group B compared to Group A (RR = 3.85, 95% CI: 1.48–10; *p* < 0.01).

#### 3.5.1 Associations With MTX Levels and Protocols

Serum MTX at 24–72 h was not associated with OM incidence or severity (Figure 4).

**FIGURE 4:**
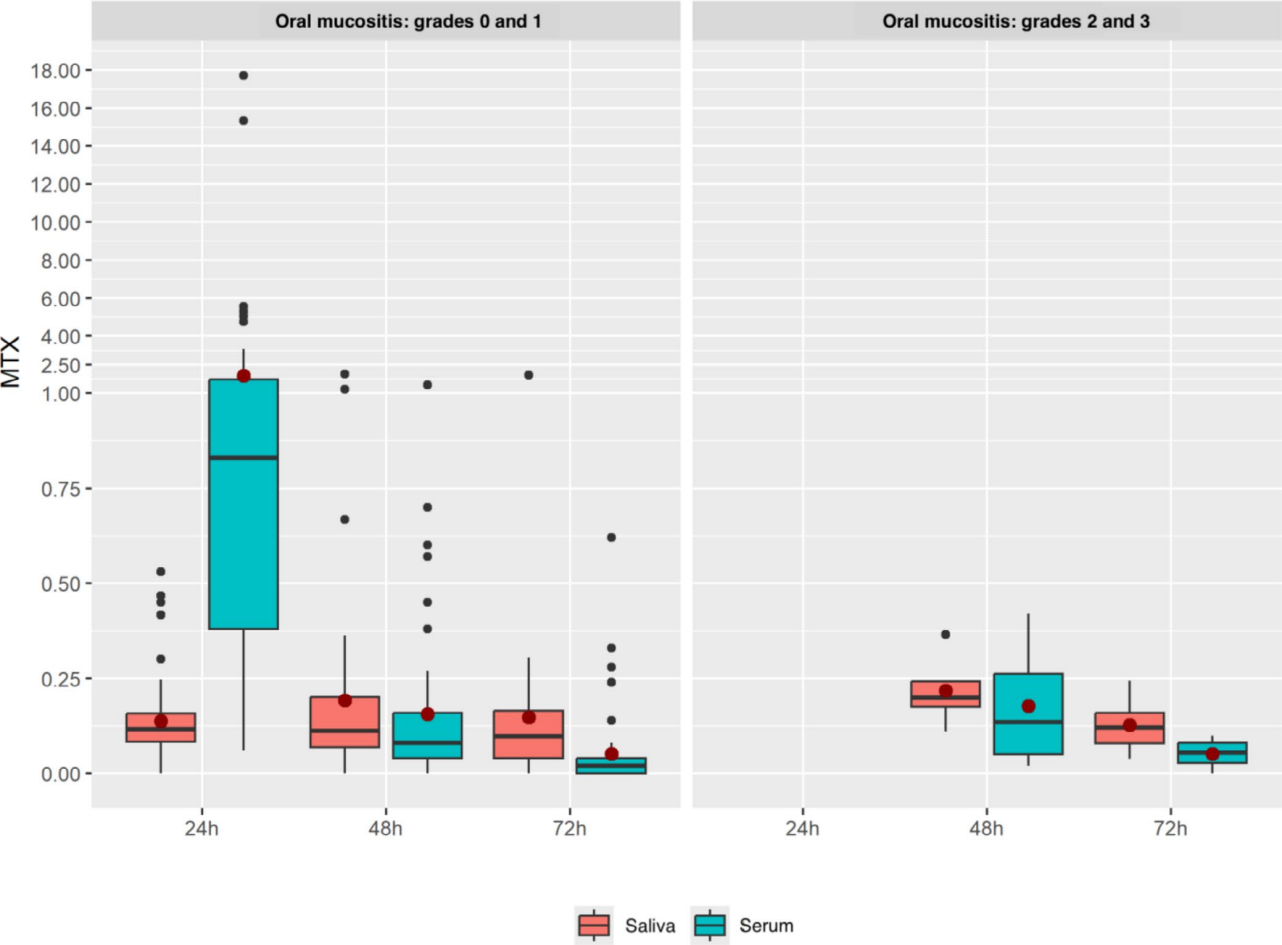
Oral mucositis grades 0 and 1, grades 2 and 3, and its correlation with serum and salivary MTX concentrations at 24 h, 48 h, and 72 h. MTX* μmol/L.

In Group A patients, the BRALLA protocol increased the risk of severe OM versus CNS prophylaxis (OR 3.64, 95% CI 1.06–12.47; *p* = 0.04). In Group B, no specific chemotherapy protocol was linked to severity, but each 500 mg increase in MTX dose increased OM risk by 9% (OR 1.09, 95% CI: 1.01–1.17; *p* = 0.02). Representative clinical images are shown in Figure S2.

### 3.6 MTX Concentration Monitoring (Serum and Saliva), pH, and OM

#### 3.6.1 Salivary MTX and OM

In Group A, each 0.01 μmol/L increase in salivary MTX at 24 h raised the odds of higher OM grades at 96 h by 12% (OR 1.12, 95% CI 1.01–1.24). In Group B, a 0.01 μmol/L increase at 48 h was associated with a 3.4% higher probability of severe OM within 9 days (OR 1.04, 95% CI 0.93–1.17).

#### 3.6.2 Urinary pH and OM

In Group A, a 1‐unit baseline pH increase raised the risk of OM at 48 h five‐fold (OR 5.04, 95% CI 1.08–23.35). In Group B, a 1‐unit pH increase at 24 h reduced the likelihood of OM at 24 h and 48 h by ~92%–93% (OR 0.07–0.08).

#### 3.6.3 Creatinine and OM

No correlation was observed between serum creatinine levels and OM development.

### 3.7 Other Toxicities (Xerostomia, Dysgeusia, Dysphagia)

Xerostomia, dysgeusia, and dysphagia data at all time points are summarized in Table S4. In Group A, no associations were found between these toxicities and OM. In Group B, grade 2 dysphagia was linked to higher OM severity compared to grades 0–1.

## 4 Discussion

This is the first prospective clinical trial in adults with hematological malignancies undergoing HD‐MTX to evaluate serum and salivary MTX concentration, OM, creatinine, urinary pH, and prophylactic PBMT.

Delayed MTX clearance increases toxicity risk, including OM (Wight et al. 2022; Howard et al. 2016; Den Hoed et al. 2015; Liu et al. 2011; Shimasaki et al. 2006). In our cohort, delayed elimination occurred in both groups, but no correlation was found between serum MTX levels at 24–72 h and OM incidence—consistent with prior pediatric studies (Valer et al. 2021; Den Hoed et al. 2015; Liu et al. 2011). Some previous studies, however, have reported an association between high or delayed MTX clearance and the development of OM in children (Rask et al. 1998; Cheng 2008).

While serum MTX is a reliable but invasive marker, salivary MTX offers a non‐invasive alternative. This rationale was first introduced by Oliff et al. (1979) who reported that salivary MTX levels were generally 1%–2% of the simultaneous serum levels and supported by Schroder et al. and Ishii et al. that found saliva levels up to 7% of the serum levels (Schrøder et al. 1987; Ishii et al. 1989). Unexpectedly, we observed much higher levels—29% at 24 h, 323% at 48 h, and 589% at 72 h—likely reflecting the free (30%–50%) MTX fraction in serum (Oliff et al. 1979; Ishii et al. 1989).

Serum MTX declined biphasically, while salivary MTX showed variable elimination with no significant correlation, consistent with prior studies (Ishii et al. 1989; Albertioni et al. 1997; Steele et al. 1979). Steele et al. (1979) observed a correlation with parotid saliva, likely due to its stable pH. In contrast, variability in mixed saliva pH may explain the inconsistent salivary levels observed (Albertioni et al. 1997).

Mucosal damage may result from direct CT contact via saliva (Epstein et al. 2002). We observed that in Group A, each 0.01 μmol/L increase in salivary MTX at 24 h raised the odds of higher OM grades at 96 h by 12%. As for Group B, a 0.01 μmol/L increase at 48 h raised risk by 3.4% within 9 days. These findings suggest that salivary MTX levels may reflect epithelial damage or local mucosal exposure to MTX. Ishii et al. (1989) observed a relationship between salivary excretion of MTX at 6 h of infusion and severity of OM, suggesting that early secretion of MTX plays a role in the development of OM. Results from Oliff et al. (1979), Albertioni et al. (1997), and Rask et al. (1996), reported no such association. This discrepancy underscores the need for standardized saliva collection protocols and consideration of timing in predictive models.

No association between serum creatinine and OM was found, unlike previous pediatric findings (Valer et al. 2021; Curra et al. 2021). Valer et al. (2021) found an association and that for each increase of one unit of serum creatinine, it was observed a 37% higher prevalence of OM in children. Curra et al. (2021) also could correlate OM and lower levels of creatinine.

In our study, OM occurred in 83.02%, with Grade 3 OM only in the placebo Group at 72 h and 96 h (7.41%). No grade 4 OM was observed. This incidence exceeds that reported in adults (6%–15%) and may reflect differences in oral care practices as well as the prospective design of our study, which included systematic daily mucosal assessments capturing all changes, including grade 1 OM, whereas most studies report only ulcerative lesions (grade ≥ 2). Therefore, our findings may reflect a more comprehensive assessment of OM rather than an overestimation of its incidence. Small sample sizes and the limited number of studies specifically evaluating MTX‐related OM in adults may also contribute to this discrepancy (Wight et al. 2022; Wilson et al. 2020; Shenoy et al. 2022). Our findings resemble those reported in pediatric populations. Importantly, demographic characteristics, comorbidities, treatment protocols, and oral care measures were comparable between groups (Table 1), minimizing the likelihood that these factors influenced the observed associations.

Baseline characteristics, including demographic variables, comorbidities, treatment protocols, oral care measures, and leukocyte count distribution, were comparable between groups (Table 1), minimizing potential confounding. Although abnormal leukocyte counts were associated with a 2.31‐fold increased likelihood of more severe OM, this did not reach statistical significance. Nevertheless, this finding may indicate a possible relationship between altered leukocyte counts and increased susceptibility to OM.

In addition, MTX‐induced OM has also been linked to alterations in the oral microbiota, and salivary immunoglobulins, proteins, electrolytes, and nonspecific host defenses in saliva (Van der Beek et al. 2019). The salivary system plays a major role in host defense, and qualitative and quantitative changes can have a profound effect on the oral mucosal tissues (Epstein et al. 2002).

Previous studies suggest that MTX‐related toxicities such as OM may be influenced by concomitant CT during HD‐MTX courses (Tsurusawa et al. 2015), and that the interpatient variability undermines the ability to accurately predict individual toxic responses using pharmacologic parameters alone (Shimasaki et al. 2006). Supporting this observation, OM risk was higher in patients receiving combined chemotherapy regimens, particularly the BRALLA protocol (vincristine, daunorubicin, pegaspargase, cyclophosphamide, cytarabine, and HD‐MTX), aligning with previous studies that found severe cases of OM to be associated with HD‐MTX + cyclophosphamide/doxorubicin and HD‐MTX combined with cyclophosphamide, and not with HD‐MTX monotherapy (Curra et al. 2021). Higher MTX doses also predicted OM; in Group B, each 500 mg increase raised OM risk by 8.84 times, consistent with pediatric findings in which OM occurred in 13% of patients receiving 5 g/m^2^, and only in 3% of patients that received 3 g/m^2^ (Shan et al. 2020).

Although the MASCC/ISOO guidelines recommend PBMT for OM prevention in HSCT and head and neck radiotherapy (with or without CT), evidence in CT settings remains limited due to the lack of randomized clinical trials and the significant variability of the PBMT parameters used (Elad et al. 2020; Zadik et al. 2019). Notably, we were unable to identify studies involving adult patients undergoing HD‐MTX and the use of PBMT, and for that reason, comparisons in this study were based on findings from pediatric populations. It is important to highlight that, the incidence of OM is higher in children than it is in adults, however, the healing process tends to be faster in the pediatric population (Heimlich et al. 2023; Calarga et al. 2024; Pels 2017; Sonis 1998).

Our study showed a significant benefit of PBMT: OM was diagnosed in 69.23% of the PBMT group versus 96.30% in the placebo group, with Grade 3 OM observed only in the latter. Group B had 8.97 times higher odds of developing higher grades of OM and nearly fourfold risk of ulcerated lesions. These results support earlier findings of Castro et al. and Nunes et al. that demonstrated that PBMT was effective in the prevention and treatment of OM, and patients that did not undergo prophylactic PBMT were at increased risk of OM (De Castro et al. 2013; Nunes et al. 2020).

Limitations include single‐center recruitment, potentially higher OM incidence due to daily assessments, and potential variability in salivary MTX measurements related to the use of whole (mixed) saliva, despite standardized collection procedures. Nevertheless, the randomized, double‐blind design and systematic monitoring support the robustness of our findings. An important consideration is that our study evaluated prophylactic PBMT, which may modulate mucosal inflammation and tissue permeability, potentially influencing salivary MTX concentrations. Thus, particularly in the PBMT group, salivary MTX should be interpreted with caution as an independent predictor of OM. Nevertheless, the randomized design with a sham control group strengthens the internal validity of our findings, and the observed association between salivary MTX and OM supports the potential of saliva as a promising and non‐invasive biomarker, warranting further investigation.

## 5 Conclusions

PBMT significantly reduced the incidence and severity of OM in adults receiving HD‐MTX, reinforcing its role as an effective preventive strategy in hematological malignancies. Although PBMT is already recommended in high‐risk settings such as hematopoietic stem cell transplantation and head and neck radiotherapy, our results suggest that similar benefits may extend to patients undergoing HD‐MTX, supporting its consideration in routine care. Further studies are warranted to refine treatment parameters and optimize implementation across oncologic settings. Serum MTX monitoring at 24, 48, and 72 h remains the standard clinical approach for toxicity assessment. However, salivary MTX showed a significant association with OM severity, indicating its potential as a complementary and non‐invasive biomarker of MTX exposure and risk. Validation in larger cohorts is needed to establish its clinical utility and to standardize PBMT‐integrated monitoring strategies.

## Author Contributions

**Ana Leticia Mores:** methodology, investigation. **Cesar Rivera:** visualization, writing – review and editing. **Carolina Guimarães Bonfim:** formal analysis, software, methodology, investigation. **Henrique Ballassini Abdalla:** methodology, formal analysis, writing – review and editing. **Iara Gonçalves Aquino:** methodology. **Juliana Trindade Clemente‐Napimoga:** methodology, writing – review and editing. **Letícia Rodrigues‐Oliveira:** conceptualization, methodology, data curation, investigation, writing – original draft. **Natalia Rangel Palmier:** methodology, investigation. **Maria Cláudia Cuzzullin:** methodology, investigation. **Rossana Veronica Mendoza López:** software, data curation, formal analysis. **Ana Carolina Prado‐Ribeiro:** conceptualization, validation, supervision, project administration, writing – review and editing, visualization, funding acquisition, resources. **Marcelo Lazzaron Lamers:** writing – review and editing, resources. **Maria Cecilia Querido de Oliveira:** methodology, writing – review and editing, investigation. **Juliana Pereira:** conceptualization, writing – review and editing. **Tatiana Natasha Toporcov:** conceptualization, writing – review and editing. **Manoela Domingues Martins:** writing – review and editing, funding acquisition, resources. **Aljomar Jose Vechiato Filho:** methodology, formal analysis, writing – review and editing. **Luiz Alcino Gueiros:** conceptualization, writing – review and editing. **Thais Bianca Brandão:** writing – review and editing, resources. **Alan Roger Santos‐Silva:** writing – review and editing, visualization, conceptualization. **Karina Morais‐Faria:** methodology, investigation. **Pablo Agustin Vargas:** resources, writing – review and editing.

## Funding

This study was supported by the Brazilian National Council for Scientific and Technological Development (CNPq), the Ministry of Science, Technology, and Innovation (MCTI), and the National Fund for Scientific and Technological Development (FNDCT) through grant No. 406291/2021‐7, as well as by the CNPq/SECTICS/CAPES/FAPs—National Institutes of Science and Technology Program (INCT), grant No. 408830/2024‐7. Alan Roger Santos‐Silva is a research grantee of the Brazilian National Council for Scientific and Technological Development (CNPq, Brazil; grant No. 304941/2020‐4) and the São Paulo Research Foundation (FAPESP; grant No. 24/20694‐1). Ana Carolina Prado‐Ribeiro is also a research grantee of the Brazilian National Council for Scientific and Technological Development (CNPq, Brazil; grant Nos. 304397/2025‐3 and 201672/2025‐1). Juliana Trindade Clemente‐Napimoga, Manoela Domingues Martins, Marcelo Lazzaron Lamers, and Pablo Agustin Vargas are also recipients of Research Productivity Fellowships from the Brazilian National Council for Scientific and Technological Development (CNPq).

## Ethics Statement

Ethical approval was obtained (CAAE: 38194420.4.0000.5418).

## Conflicts of Interest

The authors declare no conflicts of interest.

## Supporting information


**Table S1:** Photobiomodulation parameters and protocols.
**Table S2:** Salivary excretion of methotrexate (concentrations at 24 h, 48 h, and 72 h).
**Table S3:** Creatinine and pH before, at 24 h, 48 h, and 72 h.
**Table S4:** Oral toxicities and HD‐MTX at 24 h, 48 h, 72 h, 96 h, and 9 days post infusion.
**Figure S1:** CONSORT flow diagram of patient enrollment, randomization, follow‐up, and analysis.
**Figure S2:** Oral mucositis in Group A and Group B.

## Data Availability

The data that support the findings of this study are available on request from the corresponding author. The data are not publicly available due to privacy or ethical restrictions.
