# Supplementary material for: Salivary Methotrexate as a Predictor of Oral Mucositis in Hematological Cancer Patients: A Phase II Randomized, Double‐Blind Clinical Trial
Source: Oral Dis. 2026 Jul 12;32(10):3329–42. doi: 10.1111/odi.70381 (PMC13768937; doi:10.1111/odi.70381)
Supplement: Supplementary file 1 [file ODI-32-3329-s001.docx]

**Supplementary Table 1.** Photobiomodulation parameters and protocols.

| **PBMT** | **Group A** | **Group B** |
| --- | --- | --- |
| *Manufacturer* | DMC, São Carlos, SP, Brazil | DMC, São Carlos, SP, Brazil |
| Protocols | Intraoral PBMT | Placebo PBMT (using a tip that blocked light emission simulating treatment without delivering active therapy) |
| Device information | Therapy XT | Therapy XT |
| Number of emitters | Two | Two |
| Emitter type | InGaAs | InGaAs |
| Beam delivery system | Fiber optic | Fiber optic |
| *Irradiation parameters* |  |  |
| Wavelength (nm) | 660 | - |
| Spectral bandwidth (nm) | 660 ± 10 | - |
| Operating mode | Continuous wave | - |
| Radiant power (W) | 100 mW | - |
| Beam profile | Gaussian | - |
| *Treatment parameters* |  |  |
| Beam spot size at target (cm^2^) | 0.028 | - |
| Irradiance (W/cm^2^) | 3.57 | - |
| Exposure duration (s) | 10 | 10 |
| Fluence (J/cm^2^) | 35 | - |
| Radiant energy (J) | 1 | - |
| *Number of points irradiated* | 26 | 26 |
| *Application technique* | Contact | Contact |
| *Frequency* | At D0 and D1, and at D2 if plasma MTX concentrations on D1 were ≥5–10 µmol/L; at D3 if D2 concentrations were ≥1 µmol/L; and at D4 if D3 concentrations were ≥0.1 µmol/L. When concentrations were bellow threshold, PMBT was conducted with a blocking tip. | 5 consecutive days - D0, D1, D2, D3, and D4. |

PBMT Photobiomodulation therapy, InGaAs Indium-Gallium-Arsenide, J joule, cm^2^ square centimeters, nm nanometers, s second, W watts.

**Supplementary Table 2.** Salivary excretion of methotrexate (concentrations at 24h, 48h, and 72h).

| **#** | **Group A** *n=26 (49.06%)* | **Group B** *n=27 (50.94%)* | **Total** *n=53 (100%)* |
| --- | --- | --- | --- |
| **Saliva concentration** |  |  |  |
| ***24 hours*** |  |  |  |
| *≤ 0.1* | 8 (30.77) | 13 (48.15) | 21 (39.62) |
| *≤ 0.2* | 14 (53.85) | 12 (44.44) | 26 (49.06) |
| *> 0.2* | 4 (15.38) | 2 (7.41) | 6 (11.32) |
|  |  |  |  |
| ***48 hours*** |  |  |  |
| *≤ 0.1* | 13 (50) | 9 (33.33) | 22 (41.51) |
| *≤ 0.2* | 8 (30.77) | 10 (37.04) | 18 (33.96) |
| *> 0.2* | 5 (19.23) | 8 (29.63) | 13 (24.53) |
|  |  |  |  |
| ***72 hours*** |  |  |  |
| *≤ 0.1* | 14 (53.85) | 12 (44.44) | 26 (49.06) |
| *≤ 0.2* | 10 (38.46) | 10 (37.04) | 20 (37.74) |
| *> 0.2* | 2 (7.69) | 5 (18.52) | 7 (13.20) |

**Supplementary Table 3.** Creatinine and pH before, at 24h, 48h, and 72h.

|  | **Group A** *n=26 (49.06%)* | **Group B** *n=27 (50.94%)* | **Total** *n=53 (100%)* |
| --- | --- | --- | --- |
| **Creatinine** |  |  |  |
| ***Before MTX infusion*** |  |  |  |
| *Mean (Standard Deviation)* | 0.74 (0.17) | 0.81 (0.39) | 0.77 (0.30) |
| *Median (Minimum; Maximum)* | 0.73 (0.34; 1.04) | 0.71 (0.23; 2.08) | 0.71 (0.23; 2.08) |
| ***% altered*** | 6 (23.08%) | 11 (40.74%) | 17 (32.08%) |
| ***24 hours*** |  |  |  |
| *Mean (Standard Deviation)* | 0.76 (0.21) | 0.87 (0.46) | 0.81 (0.36) |
| *Median (Minimum; Maximum)* | 0.71 (0.37; 1.34) | 0.63 (0.34; 2.34) | 0.69 (0.34; 2.34) |
| ***% altered*** | 9 (34.62%) | 18 (66.67%) | 27 (50.94%) |
| ***48 hours*** |  |  |  |
| *Mean (Standard Deviation)* | 0.81 (0.27) | 0.88 (0.52) | 0.85 (0.41) |
| *Median (Minimum; Maximum)* | 0.76 (0.32; 1.47) | 0.67 (0.33; 2.22) | 0.69 (0.32; 2.22) |
| ***% altered*** | 8 (30.77%) | 19 (70.37%) | 27 (50.94%) |
| ***72 hours*** |  |  |  |
| *Mean (Standard Deviation)* | 0.80 (0.25) | 0.95 (0.58) | 0.88 (0.45) |
| *Median (Minimum; Maximum)* | 0.78 (0.28; 1.32) | 0.72 (0.23; 2.72) | 0.77 (0.23; 2.72) |
| ***% altered*** | 5 (19.23%) | 15 (55.56%) | 20 (37.74%) |
| ***pH*** |  |  |  |
| ***Before MTX infusion*** |  |  |  |
| *Mean (Standard Deviation)* | 7.58 (0.61) | 7.80 (0.54) | 7.69 (0.58) |
| *Median (Minimum; Maximum)* | 7.50 (6.50; 9.00) | 8.00 (7.00; 9.00) | 8.00 (6.50; 9.00) |
| ***24 hours*** |  |  |  |
| *Mean (Standard Deviation)* | 7.48 (0.79) | 7.78 (0.63) | 7.63 (0.72) |
| *Median (Minimum; Maximum)* | 7.75 (5.00; 8.50) | 8.00 (6.50; 8.50) | 8.00 (5.00; 8.50) |
| ***48 hours*** |  |  |  |
| *Mean (Standard Deviation)* | 7.17 (0.79) | 7.70 (0.75) | 7.44 (0.81) |
| *Median (Minimum; Maximum)* | 7.00 (6.00; 8.50) | 8.00 (6.00; 8.50) | 7.50 (6.00; 8.50) |
| ***72 hours*** |  |  |  |
| *Mean (Standard Deviation)* | 7.23 (0.90) | 7.26 (0.81) | 7.25 (0.85) |
| *Median (Minimum; Maximum)* | 7.25 (6.00; 8.50) | 7.50 (5.00; 8.50) | 7.50 (5.00; 8.50) |

**Supplementary Table 4.** Oral toxicities and HD-MTX at 24h, 48h, 72h, 96h, and 9 days post infusion.

|  | **Group A** *n=26 (49.06%)* | **Group B** *n=27 (50.94%)* | **Total** *n=53 (100%)* |
| --- | --- | --- | --- |
| **Xerostomia** |  |  |  |
| ***D0 (before MTX infusion)*** |  |  |  |
| Grade 0 | 22 (84.62%) | 26 (96.30%) | 48 (90.57%) |
| Grade 1 | 4 (15.38%) | 1 (3.70%) | 5 (9.43%) |
| ***D1 (24 hours post MTX)*** |  |  |  |
| Grade 0 | 18 (69.23%) | 22 (81.48) | 40 (75.47%) |
| Grade 1 | 7 (26.92%) | 5 (18.52%) | 12 (22.64%) |
| Grade 2 | 1 (3.85%) | 0 (0.00%) | 1 (1.89%) |
| ***D2 (48 hours post MTX)*** |  |  |  |
| Grade 0 | 14 (53.85%) | 11 (40.74%) | 25 (47.17%) |
| Grade 1 | 11 (42.31%) | 14 (51.85%) | 25 (47.17%) |
| Grade 2 | 1 (3.85%) | 2 (7.41%) | 3 (5.66%) |
| ***D3 (72 hours post MTX)*** |  |  |  |
| Grade 0 | 13 (50.00%) | 12 (44.44%) | 25 (47.17%) |
| Grade 1 | 12 (46.15%) | 12 (44.44%) | 24 (45.28%) |
| Grade 2 | 1 (3.85%) | 3 (11.11%) | 4 (7.55%) |
| ***D4 (96 hours post MTX)*** |  |  |  |
| Grade 0 | 12 (46.15%) | 16 (59.26%) | 28 (52.83%) |
| Grade 1 | 13 (50.00%) | 7 (25.93%) | 20 (37.74%) |
| Grade 2 | 1 (3.85%) | 4 (14.81%) | 5 (9.43%) |
| ***D5 (9 days post MTX)*** |  |  |  |
| Grade 0 | 20 (76.92%) | 20 (74.07%) | 40 (75.47%) |
| Grade 1 | 6 (23.08%) | 5 (18.52%) | 11 (20.75%) |
| Grade 2 | 0 (0.00%) | 2 (7.41%) | 2 (3.77%) |
| ***Dysgeusia*** |  |  |  |
| ***D0 (before MTX)*** |  |  |  |
| Grade 0 | 26 (100.00%) | 25 (92.59%) | 51 (96.23%) |
| Grade 1 | 0 (0.00%) | 2 (7.41%) | 2 (3.77%) |
| ***D1 (24 hours post MTX)*** |  |  |  |
| Grade 0 | 24 (92.31%) | 22 (81.48%) | 46 (86.79%) |
| Grade 1 | 2 (7.69%) | 5 (18.52%) | 7 (13.21%) |
| ***D2 (48 hours post MTX)*** |  |  |  |
| Grade 0 | 20 (76.92%) | 21 (77.78%) | 41 (77.36%) |
| Grade 1 | 6 (23.08%) | 6 (22.22%) | 12 (22.64%) |

**Supplementary Table 4.** Oral toxicities and HD-MTX at 24h, 48h, 72h, 96h, and 9 days post infusion.

|  | **Group A** *n=26 (49.06%)* | **Group B** *n=27 (50.94%)* | **Total** *n=53 (100%)* |
| --- | --- | --- | --- |
| **Dysgeusia** |  |  |  |
| ***D3 (72 hours post MTX)*** |  |  |  |
| Grade 0 | 17 (65.38%) | 21 (77.78%) | 38 (71.70%) |
| Grade 1 | 9 (34.62%) | 5 (18.52%) | 14 (26.42%) |
| Grade 2 | 0 (0.00%) | 1 (3.70%) | 1 (1.89%) |
| ***D4 (96 hours post MTX)*** |  |  |  |
| Grade 0 | 16 (61.54%) | 20 (74.07) | 36 (67.92%) |
| Grade 1 | 10 (38.46%) | 6 (22.22%) | 16 (30.19%) |
| Grade 2 | 0 (0.00%) | 1 (3.70%) | 1 (1.89%) |
| ***D5 (9 days post MTX)*** |  |  |  |
| Grade 0 | 24 (92.31%) | 25 (92.59%) | 49 (92.45%) |
| Grade 1 | 2 (7.69%) | 1 (3.70%) | 3 (5.66%) |
| Grade 2 | 0 (0.00%) | 1 (3.70%) | 1 (1.89%) |
| ***Disphagia*** |  |  |  |
| ***D0 (before MTX)*** |  |  |  |
| Grade 0 | 26 (100.00%) | 27 (100.00%) | 53 (100.00%) |
| ***D1 (24 hours post MTX)*** |  |  |  |
| Grade 0 | 26 (100.00%) | 27 (100.00%) | 53 (100.00%) |
| ***D2 (48 hours post MTX)*** |  |  |  |
| Grade 0 | 23 (88.46%) | 25 (59.26%) | 48 (90.57%) |
| Grade 1 | 3 (11.54%) | 2 (7.41%) | 5 (9.43%) |
| ***D3 (72 hours post MTX)*** |  |  |  |
| Grade 0 | 23 (88.46%) | 22 (81.48%) | 46 (86.79%) |
| Grade 1 | 3 (11.54%) | 3 (11.11%) | 5 (9.43%) |
| Grade 2 | 0 (0.00%) | 2 (7.41%) | 2 (3.77%) |
| ***D4 (96 hours post MTX)*** |  |  |  |
| Grade 0 | 24 (92.31%) | 22 (81.48%) | 46 (86.79%) |
| Grade 1 | 2 (7.69%) | 3 (11.11%) | 5 (9.43%) |
| Grade 2 | 0 (0.00%) | 2 (7.41%) | 2 (3.77%) |
| ***D5 (9 days post MTX)*** |  |  |  |
| Grade 0 | 26 (100.00%) | 24 (88.89%) | 50 (94.34%) |
| Grade 1 | 0 (0.00%) | 1 (3.70%) | 1 (1.89%) |
| Grade 2 | 0 (0.00%) | 2 (7.41%) | 2 (3.77%) |

**Supplementary Figure 1.** CONSORT flow diagram of patient enrollment, randomization, follow-up, and analysis.

**Supplementary Figure 2.** Oral mucositis in Group A and Group B.
